# Comment on: Perinatal exposure to a glyphosate-based herbicide impairs female reproductive outcomes and induces second-generation adverse effects in Wistar rats

**DOI:** 10.1007/s00204-018-2351-4

**Published:** 2018-11-19

**Authors:** Ian Plewis

**Affiliations:** Emeritus Professor of Social Statistics, The University of Manchester, Oxford Road, Manchester, M13 9PL UK

Dear Editors,

The article by Milesi et al. (2018) purports to show that exposing pregnant rats to glyphosate-based herbicides leads to adverse effects but only in the second generation. Unfortunately, the paper suffers from a serious methodological defect in that the authors do not appear to take account of ‘litter’ effects in their analyses. The offspring of a particular female rat are likely to be more alike than the offspring of different rats. Hence, the authors’ assumption in their statistical analyses that both the first- (F1, *n* = 75) and second-generation (F2, *n* = 482) rats are independent samples in each of their three experimental groups is incorrect. In fact, the first-generation sample is clustered by their parents (*n* = 21) and the second generation is clustered by both their parents and grandparents. Ignoring this clustering can lead to incorrect statistical inferences; in particular it is likely that the *p* values quoted in the paper are far too low, especially for the F2 rats. In other words, the differences observed at F2 could be just chance differences.

This problem appears to be widespread in the toxicology literature and needs to be taken seriously by researchers in the field. Statisticians (e.g. Goldstein 2011) have developed models—known as multilevel or hierarchical linear models—that handle these kinds of designs. It would be helpful if researchers using multi-generation designs were to make their data available in such a way that these litter effects can be quantified. Unfortunately, despite repeated requests, Milesi et al. have not done this.
